# Dynamic Characterization of Cercal Mechanosensory Hairs of Crickets

**DOI:** 10.3390/insects3041028

**Published:** 2012-10-22

**Authors:** Joel M. Book, Samuel F. Asokanthan

**Affiliations:** Department of Mechanical and Materials Engineering, The University of Western Ontario, 1151 Richmond Street, London N6A 5B9, Canada; E-Mail: jbook2@uwo.ca

**Keywords:** mechanosensory hair, cricket, experimental modal analysis, output-only modal analysis, stochastic subspace identification, laser doppler vibrometry, natural frequency, mode shape, modal damping ratio

## Abstract

Previous dynamic characterizations of the cercal mechanosensory hairs of crickets have generally been limited to the first resonant frequency and associated deflection shape. A more complete description of the mechanical dynamics of these structures could be obtained by an experimental modal analysis. This paper describes a method by which a full experimental modal analysis, giving natural frequency, mode shape, and modal damping ratio, of these sense organs can be performed. Results of this analysis, employing an unmeasured moving-air excitation and non-contact vibration measurement with an output-only identification method are presented. Two distinct types of behaviour were observed, one of which was a good match for the behaviour expected based on the literature, and one of which was quite different. These two behaviours had distinct patterns of modal parameters. The method described in this paper has been shown to be able to estimate the modal parameters, including natural frequency, modal damping ratio, and normalized mode shape, for the first mode of cercal mechanosensory hairs of crickets. The method could practically be extended to higher modes and a wide variety of other sound and vibration sense organs with the selection of appropriate excitation and specimen supports.

## 1. Introduction

The mechanical dynamics of sound and vibration sensing organs in insects are known to be of interest to both the biological community and to those involved in the design of biomimetic sensors. This is evidenced by the fact that investigations of the dynamics of a variety of these organs have been performed in the past. Some examples of these investigations include a study of hearing in parasitoid flies by Robert *et al.* [1], examination of an antennal hearing organ by Göpfert and Robert [2] and studies by Windmill *et al.* [3,4], which examined the dynamics of tympanal hearing organs in locusts and moths, respectively. One class of sensing organs which is particularly interesting is the mechanosensory hair. Investigations of the dynamics of these organs have been carried out by Kämper and Kleindienst [5] who examined response at a single point to air motions at frequencies from 10 Hz to 200 Hz, finding the frequencies of maximum displacement; Kumagai *et al.* [6], who used laser Doppler velocimetry to measure the frequency responses, also at a single point, and estimating damping factors; and Santulli *et al.* [7], who used scanning laser Doppler vibrometry to measure frequency response and deflection shape. More recently, Bathellier *et al.* [8] suggested that mechanosensory hairs might be better characterized by their frequency of maximum energy transmission efficiency, which they predicted, and found by experiments measuring hair tip velocity in response to air motion, to be higher than the maximum deflection (resonant) frequency of the hairs. They also made estimates of damping ratios, though their best frequency results are presented in an unconventional way. None of these examples present a complete modal characterization of the sensory organs studied. In the usual case, only the resonant (maximum response) frequency, and perhaps deflection shapes were presented. Damping of these sensory organs was discussed little, with only [8] addressing the subject in any real depth, although brief statements regarding estimated results were made in [2,6]. 

The primary objective of this research was to develop, validate, and present a method, using tools developed for the study of vibration in engineering contexts, whereby a complete modal description of the vibrational characteristics of an insect sound or vibration sensing organ might practically be obtained. 

The mechanosensory hairs on the cerci of crickets are known to respond to air motion, and a convenient means of generating this motion is with near-field sound using a loudspeaker. Excitation of this type has been used, for example by Kämper and Kleindienst and Kumagai *et al.* [5,6]. For measurement of response to excitation, microscanning laser Doppler vibrometry is a well-established method, commonly applied to both engineered (e.g., [9,10]) and biological (e.g., [2,3,7]) micron-scale structures. However, while applying excitation and measuring response to it are challenges for which there are well established solutions, to characterize the modal parameters of a structure conventionally requires that the excitation forces be precisely known as well. In the case of micron-scale structures such as mechanosensory hairs, this is generally difficult, if not entirely impractical. 

One method which can avoid this problem is the use of an output-only modal analysis algorithm. In this class of approaches, an unmeasured excitation, assumed to be a white noise is applied, and modal parameters estimated using only the measured response. In the present study, the Stochastic Subspace Identification method, was applied, employing a pre-existing implementation. This implementation, called MACEC [10], was originally developed for civil engineering applications at K.U. Leuven, and has been validated on both large (on the order of tens of meters, such as bridges) structures [11,12,13] and micron-scale structures, both with high natural frequencies, in the range of several kHz, as in [10], and low natural frequencies, below 50 Hz, as in [14].

The present paper describes a method, based on laser Doppler vibrometry and output-only modal analysis, by which a complete modal analysis, providing estimates of natural frequencies, mode shapes, and damping ratios for cercal mechanosensory hairs of crickets has been performed.

## 2. Materials and Methods

**Figure 1:**
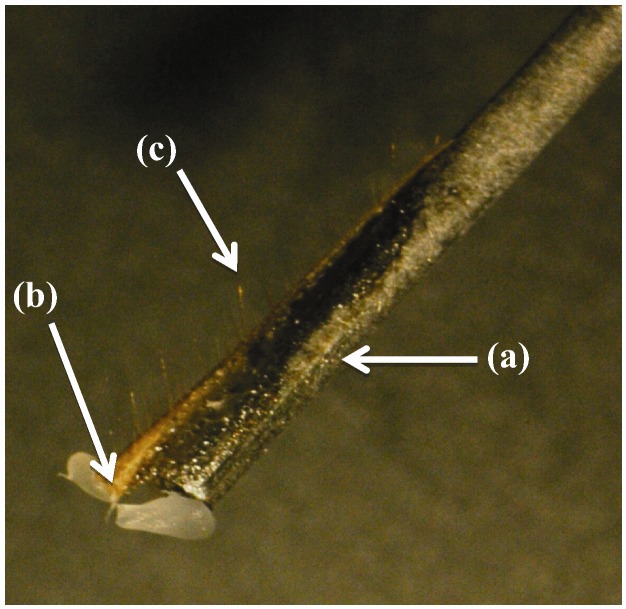
Cricket cercus attached to stiff wire; (**a**) Wire, (**b**) Cercus, (**c**) Example mechanosensory hair.

The specimens examined in this research were common feeder crickets, identified as *Acheta domestica* by their appearance. Each cricket was cooled on ice until it did not respond to tapping the container it was in. It was then pinned down and a piece of stiff piano wire was attached along the dorsal side of one cercus with cyanoacrylate glue. That cercus was then cut off, and the cut end dipped in melted wax to slow drying out. A photograph of one of the cerci examined, attached to a wire is shown in Figure 1. The wire with the attached cercus was supported so that the hairs on the ventral surface, which were expected, based on [15], to respond to transverse air motions, were visible under the vibrometer.

The method for any modal analysis can generally be described in terms of the following steps: Exciting a vibration in the structure being tested; measuring the response to this excitation; and fitting a modal model to the measurements. In most cases, using conventional modal analysis methods, the excitation must also be measured, but since this research used an output-only analysis method specifically due to the impracticality of measuring excitation applied to such small structures, that was not necessary. 

For the experiments described here, the hairs were excited using moving air, generated as near-field sound from a loudspeaker. The principal assumption in using near-field sound from a loudspeaker was that very close to the speaker, the motion of the air would be very nearly the same as the motion of the speaker cone. It is further assumed that near the center of the speaker cone this motion is approximately unidirectional along the speaker axis. These assumptions are not unreasonable within a few centimeters of the center of the speaker cone, where the cerci were positioned for these experiments. Because the first natural frequency was expected, based on [5], to be in the 50–110 Hz range, a subwoofer, specifically an Earthquake Sound SWS-8, was selected. This subwoofer was driven through a subwoofer amplifier (JBL GT5-A3001) using a band-limited white noise signal, generated as a sequence of normally distributed pseudo-random numbers, and band-pass filtered in MATLAB, with a passband of 38–250 Hz. This signal, a segment of an approximately white noise, was repeated for each measurement. The cercus was supported directly above the centre of the subwoofer under the vibrometer. The supporting structure was attached to an X-Y stage to position the cercus, allowing individual mechanosensory hairs to be positioned within the field of view of the vibrometer. The same supporting structure and excitation source had previously been used in very similar experiments performed on lengths of fine wire, detailed in [14], and good results were produced, without visible contamination by support modes.

The instrument used for measurement of the response of the hairs was a Polytec MSV-300 microscanning laser Doppler vibrometer system, consisting of a control and data acquisition PC, vibrometer and scan controllers, the vibrometer sensor itself, and a scan unit attached to a microscope. Two sets of measurements of response motion were made at each of nine points, on each of eight hairs, with lengths between 160 µm and 290 µm, on a total of six cerci. Each measurement was made with a sampling rate of 2.56 kHz, and 32,768 samples were taken. The measurements were made in a single‑point configuration, because no good location for a reference beam allowing measurement of relative velocity between the hair and the cercus was within the vibrometer’s field of view. A band‑pass filter with passband of 38–300 Hz was applied, and the measurement data was imported into the MACEC software used for the analysis, decimated by a factor of four, and detrended to remove DC bias and any motions too slow to have a complete cycle in the sample time. Some of these measurements were dominated by a few large spikes in the data, which are believed to have been caused by outside noise. These measurements were not used in the analysis.

The analysis method used here, the stochastic subspace identification algorithm, fits a modal model to measured responses to an unmeasured white noise excitation using QR factorization, singular value decomposition, and least-squares solution fitting (see, e.g., [11]). It should be noted that due to the excitation being assumed a white noise, any peaks in its spectrum will be interpreted as system modes by the identification algorithm. Further, the methods of the stochastic subspace identification algorithm assume that the system is, or at least is well approximated by, a linear system, and so is only applicable to cases where this assumption is valid. In practice, the order of model to be fit is seldom known in advance, so many models over a range of orders are fit and plotted on a stabilization diagram. The stabilization diagram, which plots each mode on axes of frequency and model order (also called system order), showing by the shape of the marker whether the modal parameters are close to those of a mode in the next lower order, is then used to select the system modes. Modes are selected manually from columns of stable modes, which are, by definition, very nearly identical regardless of the order of model they are taken from. The order of the final resulting modal model will be twice the number of selected modes. An example stabilization diagram appears in the next section. This was done for each set of measurements taken, once for each test on each hair. 

## 3. Results and Discussion

**Figure 2:**
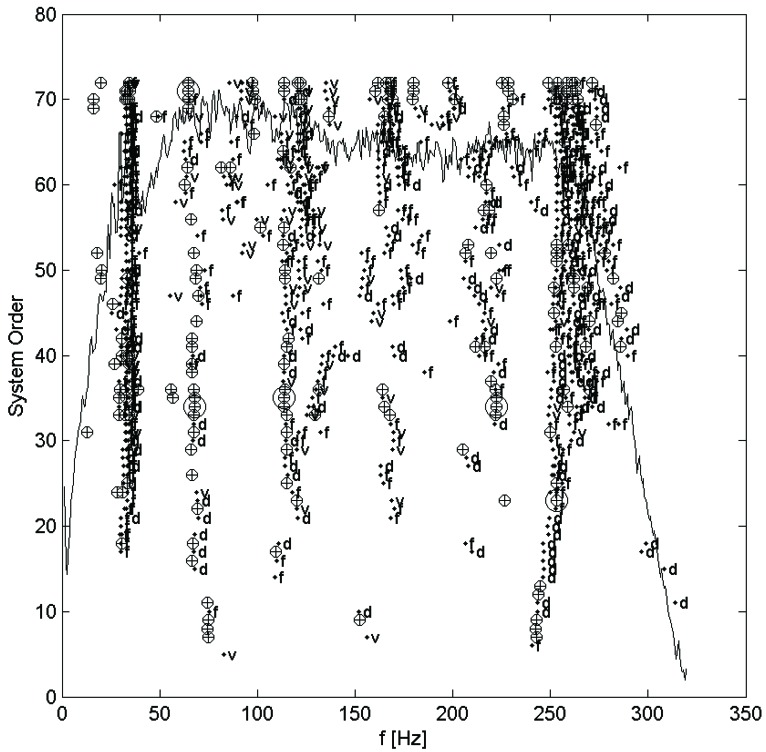
Stabilization diagram for stochastic subspace identification of a mechanosensory hair.⊕: fully stable mode; ♦: partially stable mode, with f frequency stable, d frequency and damping stable, and v frequency and mode shape stable. Figure previously appeared in [14].

**Figure 3:**
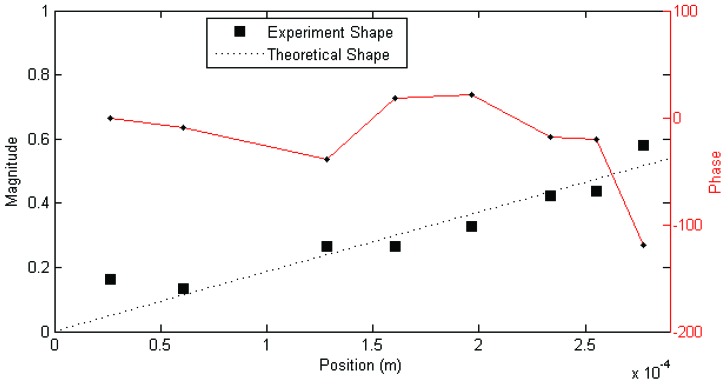
Example magnitude/phase plot of mode shape a mechanosensory hair (Hair 1, test 1; f = 72 Hz, ζ = 16%). Figure previously appeared in [14].

**Figure 4:**
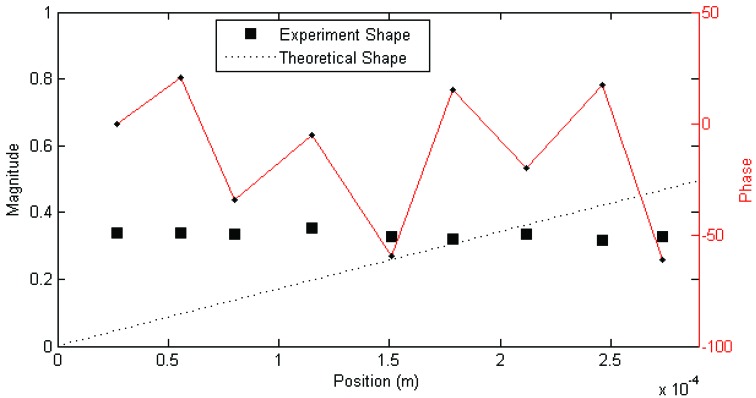
Example magnitude/phase plot of mode shape for mechanosensory hair that fits poorly to expected behaviour (Hair 4, test 2; f = 121 Hz, ζ = 7%). Figure previously appeared in [14].

**Figure 5:**
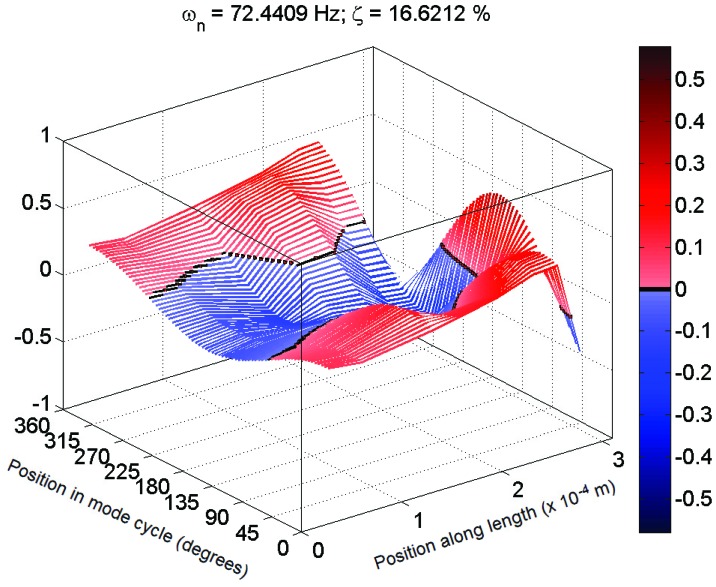
Typical 3D plot of mode shape for a mechanosensory hair following the expected behaviour in magnitude (Hair 1, test 1; f = 72 Hz, ζ = 16%).

**Figure 6:**
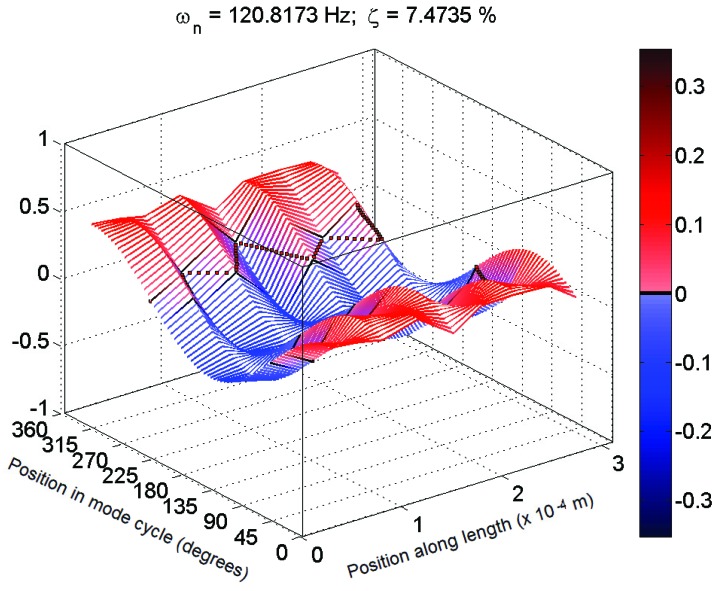
Typical 3D plot of mode shape for a mechanosensory hair that fits poorly to expected behaviour (Hair 4, test 2; f = 121 Hz, ζ = 7%).

Each set of measurements was analyzed using the MACEC implementation of the stochastic subspace identification algorithm. For each analysis, a stabilization diagram such as that shown in Figure 2 was created. In this example, two columns of stable modes, near 65 and 115 Hz, can be seen, with fully stable modes indicated by ⊕ and partially stable modes indicated by ♦. Partially stable modes are also labeled to indicate which parameters are stable, ‘f’ being frequency only, ‘d’ frequency and damping, and ‘v’ frequency and mode shape. In examination of the mode shapes estimated by the MACEC SSI implementation, the mode near 65 Hz showed a close match between the mode shape magnitude and the behaviour expected based on [5]. An example of a mode shape showing this sort of behaviour is shown in Figure 3. In other cases, modes found showed very different behaviour, such as that shown in Figure 4. These mode shapes were also plotted as motion through a full cycle of vibration. In these plots, each line represents the deflection of the structure at a specific point in its mode shape cycle. This makes it easier to visualize exactly how the structure vibrates, and to see the physical meaning of the phase variation shown in the magnitude/phase plots of Figure 3 and Figure 4. Examples are provided as Figure 5 and Figure 6, corresponding to Figure 3 and Figure 4, respectively. In all of these figures, the mode shapes are normalized, as the nature of output-only modal analysis allows only unscaled mode shapes to be obtained.

A summary of the results, including natural frequencies, damping ratios, and modal assurance criterion between observed mode shape magnitudes and expected mode shapes is presented in Table 1. In this table, bold font indicates the first dense column of stable modes appearing in the stabilization diagrams. The modal assurance criterion, or MAC, is a measure of closeness of two mode shapes, and is equal to the square of the correlation between them, varying between 0, meaning no relationship at all, and 1, where one mode shape is a simple multiple of the other. The first natural frequencies can be seen to be generally in the range of 65 to 125 Hz, which is in reasonable agreement with expectations, based on the predicted range of 50 to 110 Hz for 250 µm hairs given in [5]. The one exception, at 155 Hz, had a less dense column of stable modes near 112 Hz, which had a mode shape closer to that expected.

The complex—meaning that the mode shape vector has both real and imaginary components, in contrast to a real mode shape which has only the real components—phase behaviour of the mode shape shown in Figure 3, while possibly a reflection of the dynamics of the hairs, is actually thought to be due to the frequency of the mode being higher than the best (*i.e.*, giving the best results) frequency range of the excitation. This is because in other experiments performed previously with this excitation, a similar pattern of phase behaviour was seen, apparently depending on the frequency of the mode and best frequency range of the excitation. Details of these previous experiments may be found in [14].

**Table 1:** Summary of frequency and damping results for mechanosensory hairs, and quality of mode shape fit. Results for columns of poles are separated by “/”, and first long and dense column of stable poles is bold. Table previously appeared in [14].

**Hair/Test**	**Approximate Length (µm)**	**Frequencies (Hz)**	**Damping Ratios (%)**	**Mode Shape Fit (MAC)**
1/1	290	**72**	**16**	**0.978**
1/2		**70**/117	**17**/3	**0.956**/0.633
2/1	160	**68**/114	**17**/6	**0.953**/0.900
2/2		**69**/116	**19**/10	**0.948**/0.848
3/1	180	**120**/140	**0**/22	**0.749**/0.775
3/2		**117**/136	**4**/11	**0.846**/0.754
4/1	290	53/ **123**	31/ **3**	0.723/ **0.804**
4/2		50/ **121**	26/ **7**	0.703/ **0.754**
5/1	210	64/ **107**/120/157	11/ **16**/0/3	0.731/ **0.861**/0.869/0.875
5/2		**103**/120/157	**13**/0/3	**0.897**/0.848/0.875
6/1	180	112/ **155**	15/ **2**	0.974/ **0.925**
6/2		**111**/154	**24**/3	**0.822**/0.820
7/1	210	**75**	**21**	**0.886**
7/2		**76**	**27**	**0.908**
8/1	240	**101**/155	**13**/4	**0.822**/0.837
8/2		94/ **118**/156	10/ **1**/5	0.739/ **0.560**/0.796

**Figure 7:**
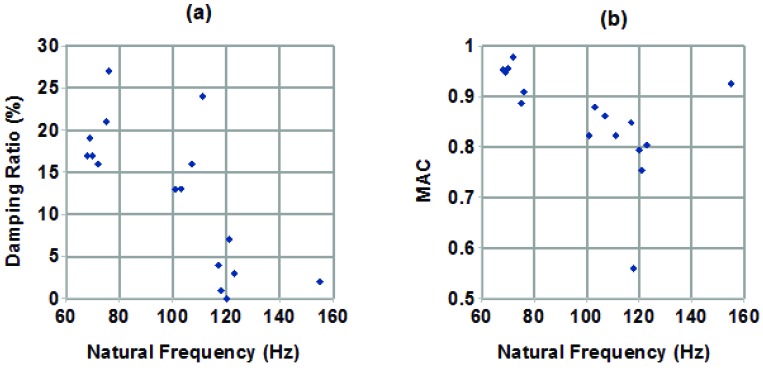
(**a**) Plot of modal damping ratio against natural frequency. (**b**) Plot of MAC relative to behaviour reported in the literature [5] against natural frequency.

Because two very different groups of mode shapes were observed, being associated with the first (lowest frequency) dense column of stable modes on the stabilization diagrams, it was deemed desirable to investigate whether there was a pattern to their appearance. No obvious strong correlation of modal parameters with hair length was seen, within the fairly narrow range of lengths examined. However, when plotting the modal parameters against each other, as in Figure 7a pattern was observed. Specifically, in Figure 7a,b, plotting damping ratio against natural frequency, and MAC against natural frequency, respectively, clusters of modes can be seen. Each point in Figure 7 represents the first mode obtained from analyzing one set of measurements, that is, there is one point in Figure 7a and one point in Figure 7b for each line in Table 1. Examining the modes in each cluster showed that there was a distinct pattern, where the modes with lower frequency tended to have higher damping and were a better match to the expected behaviour in mode shape, while those at higher frequencies tended to have lower damping and were a poorer match to the expected mode shape behaviour. The source of this second type of behaviour is not known, but possibilities include being an off-axis behaviour, where measurements were made on hairs preferentially moving in a direction other than the one in which they were excited; a mode in the cercus, rather than the hair itself, or the supporting structure; or external noise contamination. It might also simply be a mode of smaller magnitude of motion, associated with the hair or with the articulation at its base, noticed here because the expected first mode didn’t stabilize well in some cases. 

The damping estimates, which were in all cases less than one, indicating an underdamped (though quite highly damped, compared to most cases of vibration encountered in engineering contexts) mode, given by this analysis are qualitatively different from those of [6] or [8], though the cause of this discrepancy is not known. Both of these studies describe the mechanosensory hairs they examined as overdamped (damping ratio greater than 1), although [8] does give a wide uncertainty, extending well into the underdamped range, and in some cases, fairly close to the results found here. 

## 4. Conclusions

This paper describes a method by which a full modal analysis of several cercal mechanosensory hairs of crickets was carried out, giving natural frequencies, damping ratios, and mode shapes. These hairs, having lengths between 160 µm and 290 µm, were found to have natural frequencies between 65 Hz and 125 Hz in general, while damping ratios ranged from near zero to almost 30%. Examining mode shapes showed two groups of modes. The first, with natural frequencies between 65 Hz and 80 Hz and damping ratios between 15% and 30% showed a mode shape magnitude behaviour very close to that described in [5], while the second, with natural frequencies between 100 Hz and 125 Hz and damping ratios ranging from nearly zero to 24% showed a very different behaviour, not found in the literature examined. The source of this second behaviour was not determined, though a number of possibilities have been noted.

The method presented in this paper has been shown to be able to estimate modal parameters for the first mode of cercal mechanosensory hairs of crickets. This method could easily be extended to investigation of higher modes and other sound and vibration sensing organs, including two‑dimensional ones, provided that they fit into the vibrometer field of view at the magnification level needed to take measurements on them, simply by selecting a suitable excitation source and modifying the supporting structure as needed. This method offers advantages over other methods presented in the literature on sound and vibration sense organs in insects in that it does not require measurement of excitations; is relatively insensitive to environmental noise; and can provide a full modal description of the dynamics of a mechanosensory hair, or, in principal, other sound or vibration sense organ, up to the frequency limit of the excitation source.
